# Complete Blood Count Score Model Predicts Inferior Prognosis in Primary Central Nervous System Lymphoma

**DOI:** 10.3389/fonc.2021.618694

**Published:** 2021-03-26

**Authors:** Yuhua Feng, Yiping Liu, Meizuo Zhong, Leyuan Wang

**Affiliations:** ^1^Department of Oncology, The Second Xiangya Hospital, Central South University, Changsha, China; ^2^Department of Oncology, Xiangya Hospital, Central South University, Changsha, China; ^3^Department of Pediatrics, Xiangya Hospital, Central South University, Changsha, China

**Keywords:** primary central nervous system lymphoma, complete blood count, prognosis, survival, biomarker

## Abstract

**Background:**

Primary central nervous system lymphoma (PCNSL), an aggressive type of non-Hodgkin lymphoma, has a poor prognosis. Currently available prognostic scoring systems are inadequate. We therefore aimed to investigate the predictive values of complete blood counts (CBCs) in PCNSL.

**Materials and Methods:**

The cohort of this retrospective study comprised 73 PCNSL patients. The predictive values of selected CBCs, including neutrophil-to-lymphocyte ratio (NLR), platelet-to-lymphocyte ratio (PLR), systemic immune inflammation index (SII), and systemic inflammation response index (SIRI), were analyzed.

**Results:**

Ages and Memorial Sloan Kettering Cancer Center (MSKCC) scores of PCNSL patients correlated with NLR, PLR, and SII values (*p <*0.05). Both age and MSKCC scores correlated with inferior progression-free survival (PFS) and overall survival (OS) (*p <*0.05). High NLR, PLR, SII, and SIRI were significant predictors of shorter PFS and OS (*p <*0.05). NLR, PLR, SII, and SIRI were integrated to generate a “CBC score” model that accurately stratified PCNSL patients into three risk groups. The median PFS for low-risk, intermediate-risk, and high-risk groups were 24 ((12.458–35.542), 17 (10.626–23.374), and 9 (8.893–19.107) months, respectively (*p* = 0.011), and the median OS were 33 (19.175–46.825), 18 (16.368–19.632), and 9 (6.521–11.479) months, respectively (*p* = 0.008). Multivariate Cox regression model showed that MSKCC score (hazard ratio (HR) = 3.791, *p* <0.001), PLR (HR = 1.003, *p* = 0.013), and CBC score (HR = 1.873, *p* = 0.011) were independent predictors for PFS, whereas MSKCC score (HR = 4.128, *p* <0.001), PLR (HR = 1.003, *p* = 0.005), and CBC score (HR = 1.907, *p* = 0.004) were independent predictors for OS.

**Conclusion:**

The CBC score model may be a promising predictive system for PCNSL patients.

## Introduction

Primary central nervous system lymphoma (PCNSL), a rare aggressive extranodal subtype of non-Hodgkin lymphoma, is confined to the central nervous system, including the brain, eyes, spine, and pia mater (1). PCNSL represents 1–2% of all intracranial neoplasms (2) and 4–6% of all extranodal lymphomas (3). Since 2000, an increasing incidence of PCNSL has been recognized (4). Most PCNSLs are diffuse large B cell lymphoma (DLBCL), rare forms include T-cell lymphoma and Burkitt lymphoma. The prognoses of patients with PCNSL are poor, the 5-year survival rate being 33%. Without treatment, the disease progresses rapidly, the overall survival (OS) being only 1.5 months (5, 6). Identification of accurate prognostic indicators for PCNSL is urgently required.

Two scoring systems are currently used to predict the prognosis of PCNSL patients: those of the International Extranodal Lymphoma Study Group (IELSG) score and Memorial Sloan Kettering Cancer Center (MSKCC) prognostic score. The IELSG scoring system incorporates five variables, namely age, Eastern Cooperative Oncology Group performance score (ECOG PS), lactate dehydrogenase (LDH) level, cerebrospinal fluid (CSF) protein concentration and deep brain involvement (7), whereas the MSKCC scoring system uses only two variables—age and Karnofsky performance status (KPS) (8). In addition to these two scoring systems, *BCL-6* rearrangement, *6q22* deletion, *CXCL13* are reportedly associated with poor prognoses (9, 10). With the optimization of PCNSL treatment regimens, the application of molecular targeted therapy and immunotherapy, prognostic score systems for PCNSL need to be further improved.

Previous studies have shown that host immunity and systemic inflammatory responses are closely related to the development and prognosis of cancers (11, 12). The complete blood counts (CBCs) such as neutrophil-to-lymphocyte ratio (NLR) and platelet-to-lymphocyte ratio (PLR) have shown their values in predicting the prognosis of cancers. Amounts of evidences have confirmed the values of CBCs in predicting prognoses of gastric cancer (13), breast cancer (14), liver cancer (15) and lymphoma (16). However, little is known about the prognostic value of CBCs in PCNSL.

In this retrospective study, we aimed to investigate the prognostic predicting values of NLR, PLR, systemic immune inflammation index (SII) and systemic inflammation response index (SIRI) in a cohort of 73 PCNSL patients, and evaluate a new CBC score model for predicting prognoses of PCNSL patients, which hasn’t been studied before.

## Patients and Methods

### Patient Selection

A cohort of 73 patients diagnosed with PCNSL at Xiangya Hospital, Central South University from January 2012 to December 2016 was retrospectively analyzed. The inclusion criterion was that patients were pathologically confirmed PCNSL. The exclusion criteria were as follows: (1) received chemotherapy, radiotherapy or other anti-tumor therapies before diagnosed, (2) history of autoimmune diseases, (3) history of chronic inflammatory diseases such as inflammatory bowel disease, (4) with uncontrolled active infections or other illnesses.

All 73 PCNSL patients had complete clinical and follow-up data and had been followed-up from the day of diagnosis to June 2020, no patients lost to follow-up. This study was conducted in accordance with the Helsinki Declaration of 1975, revised in 2008.

### Data Collection

Clinical data including gender, age, KPS, MSKCC score, B symptoms, LDH level, and treatment regimens, were collected. Routine blood results within 1 week before initiation of therapy were also collected. The NLR, PLR, SII, SIRI were calculated as follows: NLR = neutrophil count/lymphocyte count, PLR = platelet count/lymphocyte count, SII = platelet count × neutrophil count/lymphocyte count, SIRI = neutrophil count × monocyte count/lymphocyte count. A new CBC score model incorporating these four variables was defined as follows: a score of 1 (low-risk) represents no high expression of CBCs, a score of 2 (intermediate-risk) represents 1–2 high expressions, and a score of 3 (high-risk) represents 3–4 high expressions. Overall survival (OS) was defined as the length of time from the date of diagnosis to the date of death for any cause or to the date of last follow-up; whereas progression free survival (PFS), was defined as the length of time from the date of diagnosis to the date of disease progression or death.

### Statistical Analysis

SPSS statistical software was used for data analysis (version 22.0; SPSS Inc., Chicago, IL, USA). Pearson’s χ^2^ test was used to analyze the relationships between NLR, PLR, SII, SIRI and clinicopathological features of PCNSL patients. Cut-off values of CBCs were calculated by receiver operating characteristic (ROC) curves. Survival curves were calculated according to the Kaplan–Meier method and compared using log-rank test. Multivariate analysis was based on the COX regression model. Two-sided *p* values <0.05 were considered statistical significance.

## Results

### Patient Characteristics

Relevant clinical data of the 73 PCNSL patients are summarized in **Table 1**. Fifteen of them (20.5%) were aged ≤50 years and the remaining 58 (79.5%) >50 years. 49 (67.1%) were male, and 24 (32.9%) female. 16 (21.9%) presented with elevated LDH level, and four (5.5%) presented with B symptoms. According to MSKCC score, 20.6% patients were in the “Age ≤50” group, 35.6% were in the “Age >50 and KPS ≥70” group, and 43.8% were in the “Age >50 and KPS <70” group. None of the 73 patients received any anti-tumor therapy before diagnosis, and treatment regimens were known for all patients, 18 (24.7%) had undergone chemo-radiotherapy, 53 (72.6%) surgery and chemo-radiotherapy, and two (2.7%) surgery alone.

**Table 1:** Clinicopathological characteristics of patients (n = 73).

Characteristics	Number (%)
Age	
≤50	15 (20.5%)
>50	58 (79.5%)
Gender	
Male	49 (67.1%)
Female	24 (32.9%)
LDH level	
Normal	57 (78.1%)
Elevated	16 (21.9%)
B symptoms	
Present	4 (5.5%)
Absent	69 (94.5%)
MSKCC score	
Age ≤50	15 (20.6%)
Age >50 and KPS ≥70	26 (35.6%)
Age >50 and KPS <70	32 (43.8%)
Treatment regimen	
Chemo-radiotherapy	18 (24.7%)
Surgery and chemo-radiotherapy	53 (72.6%)
Surgery	2 (2.7%)

### Optimal Cut-Off Values for CBCs and Their Correlations With Clinicopathological Characteristics

**Figure 1:**
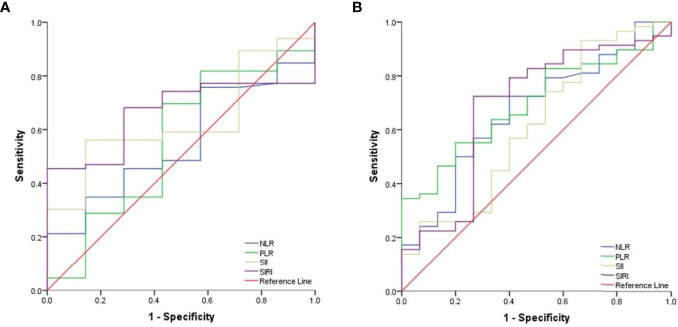
The cut-off values for CBCs. **(A)** ROC curves analysis for optimal cut-off values of NLR, PLR, SII and SIRI for PFS. **(B)** Roc curves analysis for optimal cut-off values of NLR, PLR, SII and SIRI for OS.

ROC curves were generated to calculate the optimal cut-off values for CBCs. As shown in **Figure 1A**, the optimal cut-off values for NLR, PLR, SII, and SIRI for PFS were 3.09 (area under curve (AUC) = 0.571, sensitivity = 0.8, specificity = 0.426), 152.81 (AUC = 0.674, sensitivity = 0.8, specificity = 0.574), 427.35 (AUC = 0.568, sensitivity = 1, specificity = 0.25), and 1.44 (AUC = 0.521, sensitivity = 0.8, specificity = 0.574), respectively. As shown in **Figure 1B**, the optimal cut-off values for OS were 3.09 (AUC = 0.696, sensitivity = 1, specificity = 0.435), 307.07 (AUC = 0.681, sensitivity = 0.5, specificity = 0.928), 514 (AUC = 0.652, sensitivity = 1, specificity = 0.362), and 1.44 (AUC = 0.605, sensitivity = 1, specificity = 0.58) for NLR, PLR, SII, and SIRI, respectively.

As for PCNSL patients’ clinicopathological characteristics, age and MSKCC score significantly correlated with the expressions of NLR, PLR and SII (*p* <0.05), whereas patients’ gender, LDH level, B symptoms or treatment regimens had no significant correlation with CBCs (*p* >0.05, **Table 2**).

**Table 2:** Clinicopathological characteristics according to NLR, PLR, SII and SIRI.

Characteristics	NLR high(column percentage)	NLR low(column percentage)	*p* value	PLR high(column percentage)	PLR low(column percentage)	*p* value	SII high(column percentage)	SII low(column percentage)	*p* value	SIRI high(column percentage)	SIRI low(column percentage)	*p* value
Age			0.004			0.001			0.016			0.106
≤50	4 (26.7%)	11 (73.3%)		1 (6.7%)	14 (93.3%)		8 (53.3%)	7 (46.7%)		4 (26.7%)	11 (73.3%)	
>50	39 (67.2%)	19 (32.8%)		32 (55.2%)	26 (44.8%)		48 (82.8%)	10 (17.2%)		29 (50%)	29 (50%)	
Gender			0.945			0.115			0.349			0.940
Male	29 (59.2%)	20 (40.8%)		19 (38.8%)	30 (61.2%)		36 (73.5%)	13 (26.5%)		22 (44.9%)	27 (55.1%)	
Female	14 (58.3%)	10 (41.7%)		14 (58.3%)	10 (41.7%)		20 (83.3%)	4 (16.7%)		11 (45.8%)	13 (54.2%)	
LDH level			0.741			0.483			0.627			0.315
Normal	33 (57.9%)	24 (42.1%)		27 (47.4%)	30 (52.6%)		43 (75.4%)	14 (24.6%)		24 (42.1%)	33 (57.9%)	
Elevated	10 (62.5%)	6 (37.5%)		6 (37.5%)	10 (62.5%)		13 (81.2%)	3 (18.8%)		9 (56.2%)	7 (43.8%)	
B symptoms			0.501			0.843			0.934			0.218
Present	3 (75%)	1 (25%)		2 (50%)	2 (50%)		3 (75%)	1 (25%)		3 (75%)	1 (25%)	
Absent	40 (58.0%)	29 (42.0%)		31 (44.9%)	38 (55.1%)		53 (76.8%)	16 (23.2%)		30 (43.5%)	39 (56.5%)	
MSKCC score			0.003			<0.001			0.036			0.076
Age ≤ 50	4 (26.7%)	11 (73.3%)		1 (6.7%)	14 (93.3%)		8 (53.3%)	7 (46.7%)		4 (26.7%)	11 (73.3%)	
Age>50 and KPS≥70	14 (53.8%)	12 (46.2%)		10 (38.5%)	16 (61.5%)		20 (76.9%)	6 (23.1%)		10 (38.5%)	16 (61.5%)	
Age>50 and KPS<70	25 (78.1%)	7 (21.9%)		22 (68.8%)	10 (31.2%)		28 (87.5%)	4 (12.5%)		19 (59.4%)	13 (40.6%)	
Treatment regimen			0.172			0.323			0.717			0.283
Chemo-radiotherapy	14 (77.8%)	4 (22.2%)		7 (38.9%)	11 (61.1%)		14 (77.8%)	4 (22.2%)		11 (61.1%)	7 (38.9%)	
Surgery and chemo-radiotherapy	28 (52.8%)	25 (47.2%)		26 (49.1%)	27 (50.9%)		40 (75.5%)	13 (24.5%)		21 (39.6%)	32 (60.4%)	
Surgery	1 (50%)	1 (50%)		0 (0%)	2 (100%)		2 (100%)	0 (0%)		1 (50%)	1 (50%)	

### Prognostic Factors

**Figure 3:**
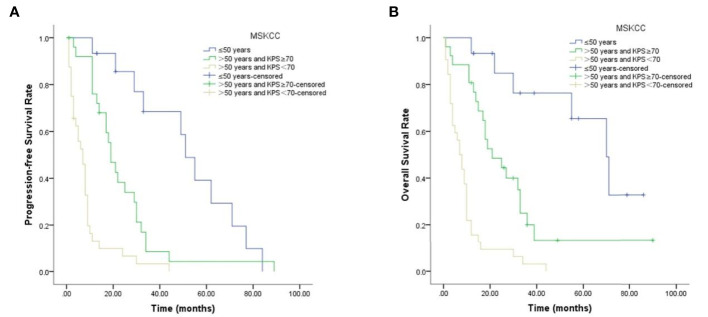
Prognostic MSKCC scores in PCNSL patients. **(A)** Results for PFS. **(B)** Results for OS.

During follow-up, 67 patients presented with disease progression, the median PFS being 13 months, and 59 patients died, the median OS being 13 months. The clinicopathological characteristics that were significantly associated with PFS and OS are shown in **Table 3**. Age and MSKCC score contributed to unfavorable predictors for PFS and OS (*p* <0.05, **Figure 3**), while patients’ gender, LDH level, B symptoms or treatment regimens had no significant correlation with prognosis (*p >*0.05).

**Table 3:** Association between clinicopathological characteristics and prognosis.

Characteristics	Median PFS (months, 95% CI)	*p* value	Median OS (months, 95% CI)	*p* value
Age		<0.001		<0.001
≤50	51 (41.963–60.037)		70 (52.903–87.097)	
>50	11 (8.845–13.155)		11 (8.514–13.486)	
Gender		0.527		0.807
Male	14 (5.667–22.333)		15 (9.216–20.784)	
Female	14 (5.784–22.216)		18 (11.060–24.940)	
LDH level		0.224		0.206
Normal	11 (6.883–15.117)		12 (6.858–17.142)	
Elevated	21 (7.120–34.880)		22 (8.428–35.572)	
B symptoms		0.494		0.399
Present	19 (not applicable)		21 (7.280–34.720)	
Absent	11 (7.502–14.498)		14 (9.138–18.862)	
MSKCC score		<0.001		<0.001
Age ≤ 50	51 (41.963–60.037)		70 (52.903–87.097)	
Age>50 and KPS≥70	19 (14.314–23.686)		21 (9.687–32.313)	
Age>50 and KPS<70	7 (4.714–9.286)		7 (4.228–9.772)	
Treatment regimen		0.932		0.902
Chemo-radiotherapy	14 (4.429–23.571)		12 (5.763–18.237)	
Surgery and chemo-radiotherapy	14 (8.064–19.936)		16 (10.382–21.618)	
Surgery	1 (not applicable)^*^		1 (not applicable)*	

^*^Sample size was not enough to calculate 95% CI of survival time.

**Figure 2:**
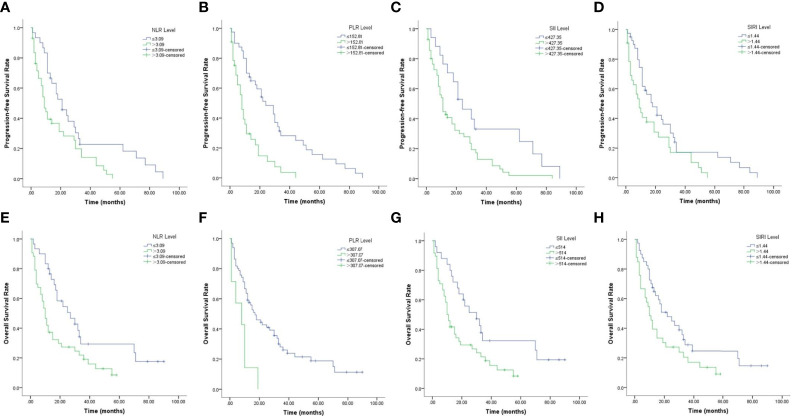
Kaplan–Meier survival curves of PCNSL patients. **(A–D)**. Kaplan–Meier curves for PFS according to NLR **(A)**, PLR **(B)**, SII **(C)** and SIRI **(D)**. **(E–H)** Kaplan–Meier curves for OS according to NLR **(E)**, PLR **(F)**, SII **(G)** and SIRI **(H)**.

Kaplan–Meier survival curves for PFS and OS with respect to CBCs are shown in **Figure 2** and **Table 4**. As shown in **Figures 2A–D**, high NLR (*p* = 0.01), high PLR (*p <*0.001), high SII (*p* = 0.012) and high SIRI (*p* = 0.024) were predictors of shorter PFS. As shown in **Figures 2E–H**, high NLR (*p* = 0.008), high PLR (*p* = 0.002), high SII (*p* = 0.01) and high SIRI (*p* = 0.037) were predictors of shorter OS.

**Table 4:** Univariate analysis of CBCs associated with PFS and OS.

CBCs	Median PFS(months, 95% CI)	*p* value	Median OS(months, 95% CI)	*p* value
NLR		0.01		0.008
High	9 (6.342–11.658)		10 (7.253–12.747)	
Low	21 (12.160–29.840)		27 (12.564–41.436)	
PLR		<0.001		0.002
High	8 (5.856–10.144)		8 (0.510–18.256)	
Low	24 (13.586–34.414)		17 (9.913–20.087)	
SII		0.012		0.01
High	11 (8.627–13.373)		10 (7.454–12.536)	
Low	24 (12.458–35.542)		30 (15.060–44.940)	
SIRI		0.024		0.037
High	9 (4.862–13.138)		10 (6.249–13.751)	
Low	18 (10.060–25.940)		22 (10.952–33.048)	
CBC score				
1	24 (12.458–35.542)	0.011	33 (19.175–46.825)	0.008
2	17 (10.626–23.374)		18 (16.368–19.632)	
3	9 (8.893–19.107)		9 (6.521–11.479)	

### The Predictive Value of CBC Score Model for Prognosis

**Figure 4:**
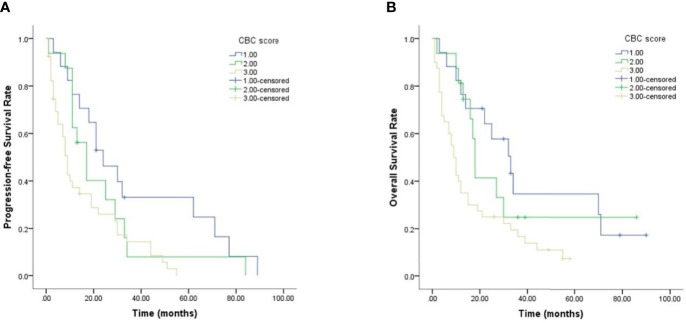
Prognostic CBC score model in PCNSL patients. **(A)** Results for PFS. **(B)** Results for OS.

Given the predictive value of NLR, PLR, SII and SIRI for PCNSL patients’ prognoses, a new CBC score model that incorporated these four variables was devised and its value in predicting prognosis evaluated. As shown in **Figure 4** and **Table 4**, PCNSL patients were divided into three risk groups. The median PFS for low-risk, intermediate-risk, and high-risk groups were 24 ((12.458–35.542), 17 (10.626–23.374), and 9 (8.893–19.107) months, respectively (*p* = 0.011), and the median OS were 33(19.175–46.825), 18 (16.368–19.632), and 9 (6.521–11.479) months, respectively (*p* = 0.008). Multivariate Cox regression analysis, including age, MSKCC score, NLR, PLR, SII, SIRI, CBC score showed that MSKCC score (hazard ratio (HR) = 3.791, *p* <0.001), PLR (HR = 1.003, *p* = 0.013), and CBC score model (HR = 1.873, *p* = 0.011) were independent predictors for PFS, and MSKCC score (HR = 4.128, *p* <0.001), PLR (HR = 1.003, *p* = 0.005), and CBC score model (HR = 1.907, *p* = 0.004) were independent predictors for OS (**Table 5**).

**Table 5:** Multivariate analysis of prognostic factors affecting PFS and OS.

Parameters	PFS			OS		
	HR	95% CI	*p* value	HR	95% CI	*p* value
Age	0.69	0.220–2.166	0.525	0.82	0.218–3.08	0.769
MSKCC score	3.791	2.041–7.042	<0.001	4.128	2.192–7.773	<0.001
NLR	1.142	0.917–1.423	0.235	1.212	0.969–1.517	0.093
PLR	1.003	1.001–1.005	0.013	1.003	1.001–1.006	0.005
SII	1	0.999–1.001	0.637	0.999	0.998–1	0.202
SIRI	0.902	0.74–1.101	0.311	0.959	0.761–1.209	0.724
CBC score	1.873	1.563–2.301	0.011	1.907	1.612–2.451	0.004

## Discussion

PCNSL is an aggressive type of non-Hodgkin lymphoma. Because it is rare, no standard treatment regimen has been established and its prognosis remains poor. In the present study, we evaluated the prognosis values of CBCs, including NLR, PLR, SII, and SIRI, in 73 PCNSL patients, and built a newly-defined CBC score model to stratify these patients into three risk groups. We found high NLR, PLR, SII, and SIRI to be closely associated with inferior PFS and OS, and that our CBC score model could be used to stratify patients with PCNSL into risk categories and predict their prognoses.

The systemic inflammatory responses are reported to be closely related to the development of various cancers, and play a key role in predicting prognosis (17). The CBCs consist of platelets, neutrophils, lymphocytes, monocytes, etc., and there is now increasing evidences that CBCs can be used to predict outcomes of cancers. The combined CBCs of the above variables, such as NLR (18), PLR (19, 20), SII (21, 22), and SIRI (23), have been reported to be useful prognostic indicators in some cancers. Furthermore, because blood cells are routinely counted, related data is easy to access, which makes CBCs promising candidates for prognostic indicators in cancers.

In the present study, we calculated optimal cut-off values for NLR, PLR, SII, and SIRI, and classified them into high- and low-level groups accordingly. In the present cohort, age and MSKCC score of 73 PCNSL patients were significantly associated with the expressions of NLR, PLR, and SII (*p <*0.05), while gender, LDH level, B symptoms or treatment regimens had no significant correlation with CBCs. In PCNSL, MSKCC score system is one of the most commonly used predicting systems in PCNSL, having been shown to enable stratification of these patients into three risk groups on the basis of age and KPS: age ≤50 years, age >50 years and KPS ≥70, or age >50 years and KPS <70. MSKCC score system is reported to be closely related to patients’ OS (8). Our findings showed age and MSKCC score, which were prognosis predictors for PCNSL, were associated with CBCs, that indicates CBCs may be related to the prognosis in PCNSL. Further analysis showed that age and MSKCC score contributed to unfavorable predictors for PFS and OS, that result was consistent with the previous study.

We also found that PCNSL patients with high NLR, PLR, SII, and SIRI had inferior PFS and OS compared with those with low NLR, PLR, SII, and SIRI. The prognostic values of NLR, PLR, SII and SIRI have been reported in many types of cancers. Xia et al. (24) analyzed the prognostic value of NLR in a cohort of 359 patients with osteosarcoma, and found that patients with NLR ≥3.43 had inferior 5-year OS and PFS, and that NLR was an independent prognostic indicator for prognosis. In PCNSL, a previous study reported that patients with NLR ≥2.0 had both worse 3-year OS (42.5% vs. 71.2%; *p* = 0.031) and 3-year PFS (37.3% vs. 60.1%; *p* = 0.028) (25). Suzuki et al. reported that comparing with lower NLR and PLR, limited-stage small-cell lung cancer patients with higher NLR and PLR had worse median and 2-year OS (NLR: 14.9 vs. 17.8 months, 29% vs. 31%; *p* = 0.026; PLR: 14.8 vs. 18.9 months, 24% vs. 37%; *p* = 0.009) (26). A retrospective analysis of 1,383 patients with colorectal cancer showed that OS and disease-free survival (DFS) were better in patients with low NLR, PLR, and SII, and that SII was an independent predictor for OS and DFS (27). Qi et al. reported that patients with advanced pancreatic cancer, who had a SIRI ≥1.8 had shorter time to progression and shorter OS than those with lower SIRI, and multivariate analysis confirmed that SIRI was an independent prognostic factor for both TTP and OS (28). The above results were consistent with ours, indicating that CBCs including NLR, PLR, SII, and SIRI, may be prognostic factors for PCNSL patients.

We defined a new CBC score model and evaluated its prognostic value in PCNSL patients. We found that the median PFS for low-risk, intermediate-risk and high-risk groups were 24 ((12.458–35.542), 17 (10.626–23.374), and 9 (8.893–19.107) months, respectively (*p* = 0.011), and the median OS were 33 (19.175–46.825), 18 (16.368–19.632), and 9 (6.521–11.479) months, respectively (*p* = 0.008), indicating that patients in the high-risk group had a significantly inferior prognosis. In a previous retrospective study of adults with T-lymphoblastic lymphoma, a similar CBC score model consisting of lymphocyte-monocyte ratio, NLR, and PLR was evaluated, and it was found that the median PFS for the low-risk, intermediate-risk, and high-risk groups was not reached, 16 months, and 7 months, respectively (*p* = 0.004), whereas the median OS for these three groups was not reached, 46 months, and 20 months, respectively (*p* = 0.007) (29). These findings were consistent with ours, indicating that CBC score model is valuable in predicting PCNSL patient’s prognosis.

This study had several limitations. First, it was a small, retrospective, single center study. Second, the correlation between specific treatment regimens or treatment responses with prognosis was not validated. Third, the CBCs biomarkers could be influenced by various unidentified factors, such as immune status, which could bring bias to results. Further studies are needed to confirm the role of CBCs in PCNSL patients.

In summary, in the present study we showed that CBCs including NLR, PLR, SII, and SIRI can serve as prognostic indicators in PCNSL patients, and that our newly-defined CBC score model may be a promising predictive score system for PCNSL patients.

## Data Availability Statement

The original contributions presented in the study are included in the article/supplementary material. Further inquiries can be directed to the corresponding author.

## Ethics Statement

The studies involving human participants were reviewed and approved by the Ethics Committee of Xiangya Hospital of Central South University. The patients/participants provided their written informed consent to participate in this study.

## Author Contributions

LW designed the research study and collected the clinical data. YF performed the research and drafted the manuscript. YL and MZ participated in the literature search and analyzed the data. All authors contributed to the article and approved the submitted version.

## Conflict of Interest

The authors declare that the research was conducted in the absence of any commercial or financial relationships that could be construed as a potential conflict of interest.
